# Resolutions of New York State Society

**Published:** 1893-12

**Authors:** John L. Moffat

**Affiliations:** Secretary; Brooklyn, N. Y.


### RESOLUTIONS OF NEW YORK STATE SOCIETY.

At the forty-second semi-annual meeting of the New York State Homœopathic Medical Society, October 4th, 1892, it was unanimously

*Resolved*, That all educated physicians should have the fullest possible knowledge of drug action, without distinction of school or creed, and that this instruction should be comprised in the curriculum of every medical college.

*Resolved*, Until all students are so taught it is manifestly unfair to require by law of candidates for licensure an examination in materia medica and therapeutics other than in accordance with the tenets of the school to which they belong.

*Resolved*, While we strongly urge upon all homœopathic colleges the necessity of giving to students the broadest and most liberal education, until the other schools of medicine shall so fully and completely teach materia medica and therapeutics we cannot submit our students to unjust discrimination in State medical examinations.

*Resolved*, That we cordially indorse the present system of licensure, and are opposed to any modification of it.

*Resolved*, That a copy of these resolutions be sent to each homœopathic college, to each homœopathic journal, to our Board of Medical Examiners, and to the Regents.

BROOKLYN, N. Y., Oct. 22d, 1893. JOHN L. MOFFAT, *Secretary*.

